# Fission Yeast Hotspot Sequence Motifs Are Also Active in Budding Yeast

**DOI:** 10.1371/journal.pone.0053090

**Published:** 2012-12-31

**Authors:** Walter W. Steiner, Estelle M. Steiner

**Affiliations:** 1 Department of Biology, Niagara University, Lewiston, New York, United States of America; 2 Science and Technology Division, Niagara County Community College, Sanborn, New York, United States of America; University of Cambridge, United Kingdom

## Abstract

In most organisms, including humans, meiotic recombination occurs preferentially at a limited number of sites in the genome known as hotspots. There has been substantial progress recently in elucidating the factors determining the location of meiotic recombination hotspots, and it is becoming clear that simple sequence motifs play a significant role. In *S. pombe*, there are at least five unique sequence motifs that have been shown to produce hotspots of recombination, and it is likely that there are more. In *S. cerevisiae*, simple sequence motifs have also been shown to produce hotspots or show significant correlations with hotspots. Some of the hotspot motifs in both yeasts are known or suspected to bind transcription factors (TFs), which are required for the activity of those hotspots. Here we show that four of the five hotspot motifs identified in *S. pombe* also create hotspots in the distantly related budding yeast *S. cerevisiae.* For one of these hotspots, *M26* (also called *CRE*), we identify TFs, *Cst6* and *Sko1*, that activate and inhibit the hotspot, respectively. In addition, two of the hotspot motifs show significant correlations with naturally occurring hotspots. The conservation of these hotspots between the distantly related fission and budding yeasts suggests that these sequence motifs, and others yet to be discovered, may function widely as hotspots in many diverse organisms.

## Introduction

Meiosis is the process of forming haploid cells (spores or gametes) from diploid cells and occurs in all sexually-reproducing organisms. It is accomplished by two successive cell divisions following a single round of DNA replication. Prior to the first meiotic division, recombination usually occurs between homologous chromosomes. This recombination shuffles alleles between maternal and paternal homologs, which serves to maintain genetic diversity in a population. Crossover recombination also forms connections between homologs, which is required in most organisms for the proper segregation of homologous chromosomes at the first meiotic division [1].

Meiotic recombination is initiated by the formation of double-strand DNA breaks (DSBs), which can be repaired using either a sister chromatid or homologous chromosome as a template [2], only the latter of which gives rise to genetically observable recombination events. DSBs are not uniformly distributed across the chromosomes of most organisms, but occur preferentially at a limited number of sites known as hotspots. Recombination hotspots have been intensively studied in recent years, and the factors determining their distribution in the genome are now emerging. White et al. showed that a hotspot in the promoter of *HIS4* requires the transcription factors (TFs) Bas1, Bas2, and Rap1 for hotspot activity [3]. The requirement for specific transcription factors also implied the involvement of specific sequence motifs that are recognized by these factors. Later it was shown that Bas1 was involved in the formation of DSBs when bound to its target sequence, TGACTC, at some sites in the genome but not others [4], [5]. In the distantly related fission yeast *S. pombe*, systematic mutagenesis revealed that a simple sequence, ATGACGT, was necessary for high levels of recombination at the *ade6-M26* hotspot [6], which also requires the Atf1-Pcr1 transcription factor for activity [7], indicating that the involvement of TFs may be a widely conserved feature of meiotic recombination hotspots.

The phenomenon of sequence-dependent hotspots of recombination has attracted increased interest recently with the discovery that a 13 bp degenerate motif is responsible for up to 40% of human hotspots [8]. That motif, CCNCCNTNNCCNC, is bound by the PRDM9 zinc finger protein that trimethylates lysine 4 of histone H3 (H3K4) [9]. H3K4 trimethylation is also required for high-level DSB formation at the majority of hotspots in *S. cerevisiae* [10], though no similar observation has been reported for *S. pombe*. In humans, PRDM9 affects recombination not only at sites containing its target sequence, but also those lacking an obvious binding site [11], suggesting that PRDM9 affects recombination both directly, by binding at hotspots, and indirectly, by an as yet unknown mechanism, that is unlikely to include direct binding [12]. Therefore, it is possible that the majority of human hotspots are determined by factors other than, or in addition to, PRDM9. These other determinants may include other sequence motifs.

Global analyses of meiotic DSB distributions in both the fission and budding yeasts revealed that the majority of DSBs occur in intergenic regions [5], [13]–[15]. Since these regions contain promoters, where transcription factors bind to regulate the expression of neighboring genes, this observation is consistent with the hypothesis that many hotspots require specific nucleotide sequences. This model is also consistent with a recent study showing that over 50% of DSBs are located within 500 bp of confirmed TF binding sites [5]. Further, at least 30% of DSBs in that study were centered on a TF binding site, suggesting these factors play an integral role in directing Spo11, the protein that forms meiotic DSBs, to those sites. DSBs centered on TF binding sites were categorized as class 1, 2, or 3 depending on whether they showed strong, weak, or no occlusion of DSBs, respectively, around the TF binding site itself (Table S3 in [5]). Though few of the TF binding sites analyzed were completely predictive of DSBs, they are certainly among one of several factors, including local and regional chromatin structure, that determine the location of hotspots. Whether they play a causative role in the majority of hotspots has yet to be determined.

Previously, we showed that a large number of different sequences are capable of generating recombination hotspots in *S. pombe* [16]. For example, ∼0.6% of random 15 bp sequences produced hotspots in the *ade6* gene. Assuming that the entire 15 bp sequence is not required for activity, we concluded that approximately 10 seven-bp motifs, or a larger number of eight- or nine-bp motifs, could account for this high frequency. Among the ∼500 sequences that produced hotspots, we identified five families of hotspots ≤10 bp in length that occurred multiple times, including the previously identified *CRE* family of hotspots [17]. Each of these sequences produced a hotspot when reconstructed by minimal base changes to the wild-type *ade6* sequence. We also identified transcription factors required for activity of hotspots representing two of these families [18] (not including the *CRE* family, which is already known to require the Atf1-Pcr1 transcription factor). Based on these results, we proposed that simple sequence motifs could account for the majority, or possibly all, hotspots in *S. pombe* and perhaps other organisms. This model was expounded upon by Wahls and Davidson [19], who also noted that our hypothesis could help to resolve the so-called hotspot paradox [20] and account for the evolutionarily rapid redistribution of hotspots that has occurred, for example, between chimpanzees and humans [21].

The question addressed in this study is whether hotspot motifs are organism-specific or conserved across species. Here we show by direct test that four of five hotspots identified in the fission yeast are also active in the budding yeast. Given that these two yeasts are considered as evolutionarily divergent from each other as either is to humans [22], our results suggest that there may be a universal catalog of sequence motifs capable of producing hotspots in most organisms.

## Results and Discussion

### Fission Yeast Hotspots are Active in Budding Yeast

Previously, we identified five families of sequence motifs that produced hotspots of recombination in the fission yeast *S. pombe*. These families were grouped based on sequence similarity and termed *CRE*, *CCAAT*, *oligo-C*, *4095*, and *4156* (The last two are *ade6* allele numbers. *4156* was referred to as motif 8–6 in [16]). The *CRE* family of hotspots includes the well-characterized *M26* hotspot [23], which is how we will refer to it in the remainder of this paper. The sequences of the motifs we tested are as follows: *M26* (ATGACGTCAT), *CCAAT* (CCAATCA), *oligo-C* (CCCCGCAC), *4095* (GGTCTRGAC), and *4156* (WNTCGGCCGA). For some motifs, we included up to five additional bp substitutions or insertions flanking the motif that our previous analyses suggested may contribute to hotspot activity (Table S1; [16], [18]). Control alleles were created for each motif that contained the same number and types of nucleotide substitutions (Table 1 and Table S1).

**Table 1:** *ADE2* alleles.

ADE2allele	Strain numbers	description^a^	Location^b^
1003	Wsc72, 126, 143,172, 183	3′-stop	ORF
1006	Wsc64	*M26*	ORF
1007	Wsc66	5′-stop	ORF
1008	Wsc70, 127, 157, 179,184	3x-*M26*	ORF
1014	Wsc92	*CCAAT*	ORF
1015	Wsc94	*Oligo-C*	ORF
1016	Wsc97	*4095*	ORF
1017	Wsc103	*4156*	ORF
1021	Wsc110, 202	*M26* (no 5′-stop)	promoter
1025	Wsc115, 129, 144,173, 186	*M26*	promoter
1026	Wsc116, 130, 145,187, 200	*M26* control	promoter
1027	Wsc117	*4156*	promoter
1028	Wsc118	*CCAAT*	promoter
1030	Wsc120, 128, 174,185, 197	3x-*M26* control	ORF
1031	Wsc121	*4095*	promoter
1032	Wsc125	*oligo-C*	promoter
1034	Wsc134	*4156* control	promoter
1035	Wsc135	*oligo-C* control	promoter
1036	Wsc136	*oligo-C* control	ORF
1038	Wsc156	*4095* control	promoter
1047	Wsc166	Bas1/Reb1 BSKO^c^(no stop)	promoter
1048	Wsc167	*CCAAT* control	promoter
1049	Wsc168	Bas1/Reb1 BSKO^c^	promoter
1050	Wsc169	*M26* control	ORF

a Name of hotspot motifs and control alleles in *ADE2* gene. For complete details of each *ADE2* allele and strains, see supplementary Table S1.

b Indicated motifs and controls were located either in the *ADE2* open reading frame (ORF) or promoter.

c Binding Site KnockOuts.

**Figure 1:**
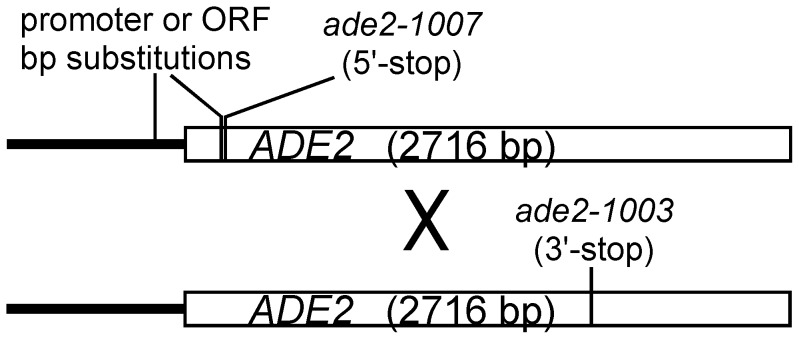
Schematic representation of the *ADE2* coding region (open rectangles) and promoter (thick black line) and the positions of sequence substitutions tested in this study. Crosses were performed with the indicated allele, *ade2-1003* (3′-stop) to determine recombination frequencies of sequence motifs created in the promoter or 5′-region of the ORF.

In order to test for potential hotspot activity, we introduced each motif into the *ADE2* gene, either in the open reading frame (ORF) or in the gene promoter (Fig. 1). We chose the *ADE2* gene because recombination within *ADE2* can be readily scored and measured by growth on the appropriate media. In addition, the red colony phenotype of *ade2* mutants has genetic advantages for future studies, including the potential to screen for additional sequences that create hotspots [16]. *ADE2* is an *S. cerevisiae* ortholog of *ade6* in *S. pombe*, where the *M26* hotspot was first identified, though we did not necessarily expect any additional similarities regarding the behavior of hotspots in that region.

**Figure 2:**
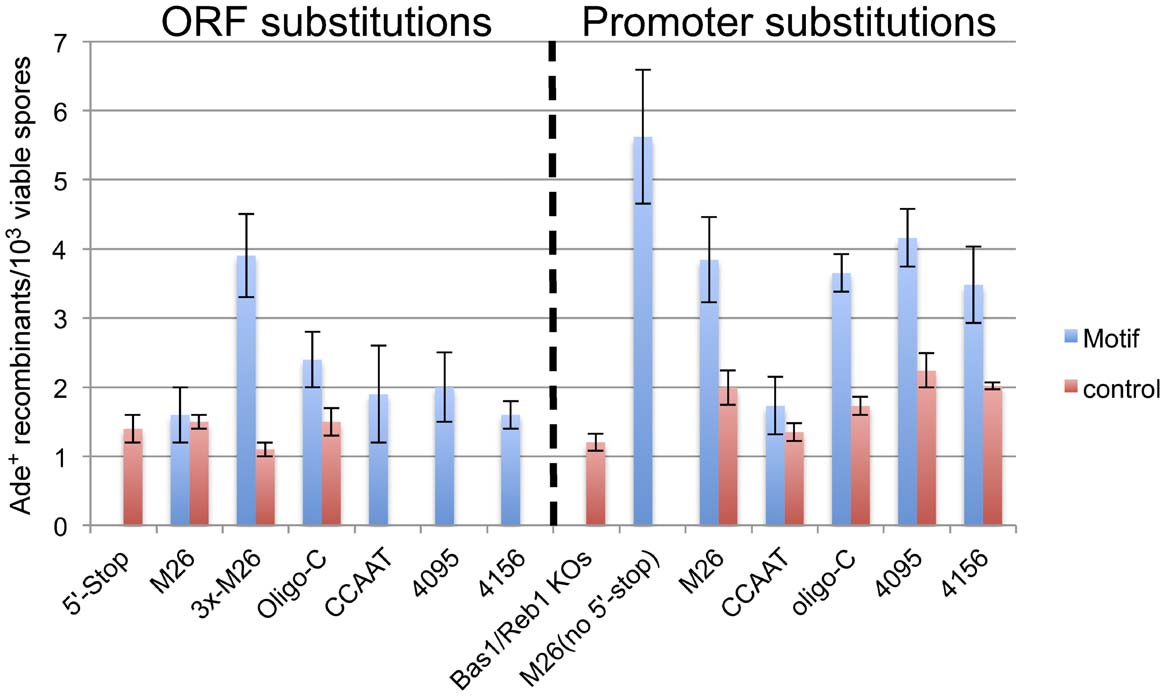
Most *S. pombe* hotspot motifs are active in the *ADE2* gene of *S. cerevisiae.* Each of the indicated motifs was tested in the *ADE2* coding sequence (ORF substitutions; left), or promoter (promoter substitutions; right). Bars indicate the mean frequency of Ade^+^ recombinants from at least three crosses of the indicated motif (blue) and its corresponding control (red) when crossed with Wsc72 (*ade2-1003*) . Error bars indicate one standard deviation. Strains crossed to Wsc72 are from left to right: Wsc66 (5′-Stop; *ade2-1007*), Wsc64 and 169 (*M26* and control; *ade2-1006* and *-1050*), Wsc70 and 120 (3x-*M26* and control; *ade2-1008* and *-1030*), Wsc94 and 136 (*Oligo-C* and control; *ade2-1015* and *-1036*), Wsc92 (*CCAAT*; *ade2-1014*), Wsc97 (*4095; ade2-1016*), Wsc103 (*4156; ade2-1017*), Wsc168 (Bas1/Reb1 KOs; *ade2-1049*), Wsc110 (*M26* no 5′-stop; *ade2-1021*), Wsc115 and 116 (*M26* and control; *ade2-1025* and *-1026*), Wsc118 and 167 (*CCAAT* and control; *ade2-1028* and *-1048*), Wsc125 and 135 (*Oligo-C* and control; *ade2-1032* and *-1035*), Wsc121 and 156 (*4095* and control; *ade2-1031* and *ade2-1038*), Wsc117 and 134 (*4156* and control; *ade2-1027* and *-1034*).

We initially tested for potential hotspot activity of the five motifs mentioned above by placing them near the 5′ end of the *ADE2* coding region and performing crosses to a strain containing a stop mutation approximately 1 kb away (*ade2-1003*, Fig. 1 and Table S1). As a baseline for recombination in this gene, we used another stop mutation, *ade2-1007* (5′-stop), located close to the sites of the tested motifs. Of the five motifs tested, only the *oligo-C* hotspot (*ade2-1015*) showed a significantly higher frequency of recombination than either of the control alleles, *ade2-1007* (P<0.002, t-test) or *ade2-1036* (p<0.04, t-test; Fig. 2), which contains the same number and type of mutations. Since the other motifs did not significantly elevate recombination compared to the *ade2-1007* (5′-stop) allele, we did not test additional sequence-matched controls. Although the individual motifs showed little or no significant hotspot activity, we noted that three tandem repeats of the *M26* hotspot (*ade2-1008*) elevated recombination approximately 4-fold relative to a sequence-matched control allele (*ade2-1030*) containing 3 tandem repeats of a different sequence with identical nucleotide composition (Table S1). Thus, the *M26* hotspot is functional in the *ADE2* ORF, but more than one copy may be required to overcome whatever factors otherwise restrict hotspot activity in this region.

**Figure 3:**
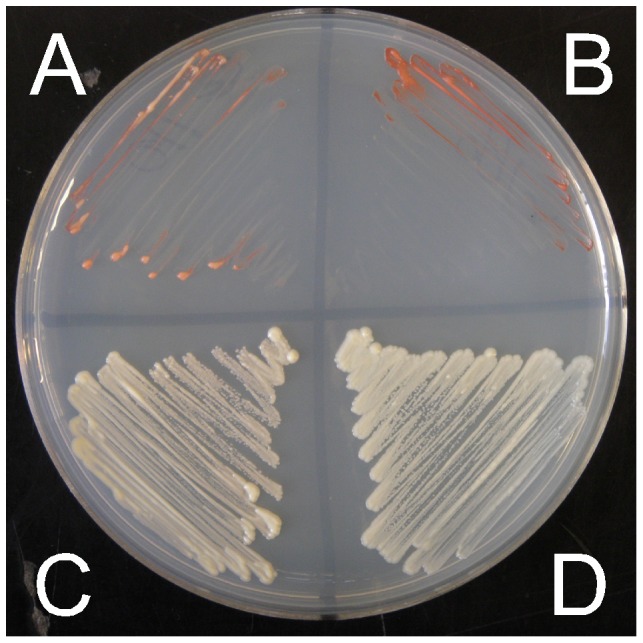
*ADE2* expression requires native transcription factor binding sites, and is repressed by the *M26* motif in *sko1^+^*. Growth of strains on NBA dropout medium lacking adenine after two days at 30°C. A) Wsc110; *ade2-1021* (*M26* no 5′-stop), B) Wsc166; *ade2-1047* (Bas1/Reb1 KOs), C) Wsc202; *ade2-1021* (*M26*-no 5′-stop *sko1Δ*), D) Wsc24; *ade2^+^*.

**Figure 4:**
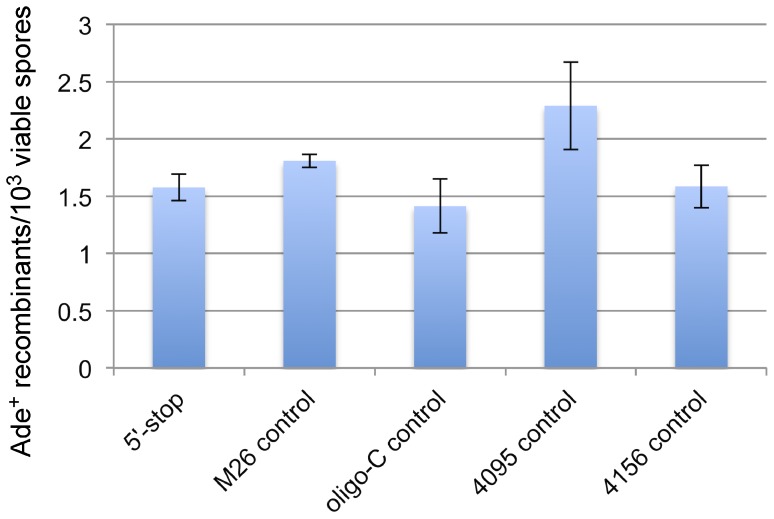
Two control alleles show significantly higher recombination than *ade2-1007* (5′-stop). Control alleles containing promoter mutations were tested side-by-side with the *ade2-1007* (5′-stop) allele to determine whether the elevated recombination observed in some of these controls (Fig. 2) is real or due to day-to-day variation. Bars indicate the mean frequency of recombinants from four or five crosses. Error bars indicate one standard deviation. Strains crossed to Wsc72 are from left to right: Wsc66 (5′-Stop; *ade2-1007*), Wsc116 (*M26* control; *ade2-1026*), Wsc135 (*Oligo-C* control; *ade2-1035*), Wsc156 (*4095* control; *ade2-1038*), Wsc134 (*4156* control; *ade2-1034*).

Given that 88% of DSB hotspots in *S. cerevisiae* overlap with gene promoters [5], it is possible that these regions are more permissive for the activity of potential hotspot motifs. Therefore, we also tested whether any of the five hotspot motifs showed activity when placed in the *ade2* promoter approximately 200 bp upstream of the *ade2-1007* (5′-stop) mutation (Fig. 1). We placed each motif and their controls in place of the TATA box, suspecting this might produce adenine auxotrophs without the need for an additional ORF mutation. However, only the *M26* motif produced an Ade^−^ phenotype (Fig. 3). Therefore, we tested the activity of the promoter motifs in conjunction with the *ade2-1007* (5′-stop) mutation, which would likely co-convert at high frequency with a closely linked hotspot [24]. When located in the *ADE2* promoter, four of the five motifs that produce hotspots in *S. pombe* also result in significantly higher levels of recombination than their respective controls (P values 0.003 – 0.0006). Surprisingly, several of the control alleles (*M26, 4095*, and *4156*) also produced modestly, but significantly higher levels of recombination than the *ade2-1007* (5′-stop) mutation alone, confirming the importance of measuring each motif against closely matched control alleles. In order to determine whether the modest increases in recombination produced by these control alleles were legitimate or, instead, due to day-to-day variation, they were tested side-by-side with the *ade2-1007* (5′-stop) mutation (Fig. 4). Day-to-day fluctuations in recombination frequencies in identical crosses have been observed in *S. pombe* [6] [25], and may also occur in *S. cerevisiae*, possibly due to subtle differences in growth or sporulation conditions for crosses performed at different times.

In side-by-side crosses, only the *M26* and *4095* control alleles continued to show significantly higher levels of recombination than the *ade2-1007* (5′-stop) allele alone. It has been shown previously that large insertions of foreign DNA in *S. cerevisiae* can create hotspots of recombination [26]–[28]. Our data suggest that even limited, and presumably random, sequence changes may affect recombination frequencies, albeit modestly. However, the hotspots created by the specific motifs tested here are unlikely to be due simply to the introduction of "foreign" DNA. If it were, then each control allele would have a 50% chance of being hotter than its associated motif. For the six pairs in which at least one of the alleles created a hotspot, the tested motif was hotter than the control allele in each of the six cases (Fig. 2). The probability of this occurring by chance is 0.5^6^ = 0.016.

Since substitution of *M26* for the *ade2* TATA box (*ade2-1021*) produced an Ade^−^ phenotype by itself, we also tested the frequency of Ade^+^ recombinants resulting from crosses using this allele, which would not require co-conversion of the *ade2-1007* (5′-stop) mutation in the ORF. Figure 2 shows that the frequency of Ade^+^ recombinants is significantly higher in the absence of the nearby 5′-stop mutation (Promoter substitutions, *M26* no 5′-stop vs. *M26*; P<0.002; t-test), indicating that these markers often fail to co-convert. Thus, the stimulatory effect of the *M26* hotspot, and probably the other hotspots, may be greater than indicated by the data in Figure 2 .

Of the five hotspot motifs, only the CCAAT motif failed to significantly elevate recombination compared to its control allele. We previously noted that this same motif in *S. pombe* was sensitive to nucleotide changes at least 6 bp (and possibly more) from the core CCAATCA sequence [18], and it is possible that a similar phenomenon is occurring here. Nevertheless, it is likely that the CCAATCA motif does create a hotspot in at least some contexts in *S. cerevisiae*, because more than 50% of those sites shown to bind the CCAAT-binding factor (CBF) are associated with hotspots in that organism [5]. In addition, this motif was categorized as class 1, meaning that the associated DSBs are actually centered on either side of the HAP2-3-4-5 (CBF) binding site, with fewer DSBs at the binding site itself likely being due to the occlusion of Spo11. A similar phenomenon was observed for the *M26* hotspot in *S. pombe* [29], where involvement of the Atf1-Pcr1 transcription factor in hotspot activity has been demonstrated unambiguously [7], [30].

### Two Hotspot Motifs are Significantly Correlated with Meiotic DSB Sites

Since the hotspots we created in the *ade2* gene resulted from mutations, we also tested whether any of these sequence motifs showed significant correlations with the positions of natural meiotic DSBs as described in [5] (Table 2). The *M26*, *CCAATCA*, and *4095* motifs did not show a significant correlation with DSBs. In fact, the *CCAATCA* motif was significantly underrepresented in DSBs. However, this underrepresentation may be due to the significant underrepresentation of this motif in intergenic DNA (Table 2), where the vast majority of meiotic DSBs are found [5]. Although we tested a 10 bp *M26* motif, because of its very high hotspot activity in *S. pombe* [25], this motif was originally shown to require only a 7 bp sequence, ATGACGT [6]. This shorter version is more abundant in the *S. cerevisiae* genome, but also shows no significant association with DSBs (Table 2). The lack of correlation of DSBs with the *M26* and *CCAATCA* motifs does not necessarily mean these motifs are not active anywhere in the *S. cerevisiae* genome, but the number of active hotspots, if any, is not sufficient to show statistical significance. In addition, as mentioned above, more than half of CBF binding sites lie within hotspots [5]. The lack of significant association between DSBs and either the *M26* and *CCAATCA* motifs is in stark contrast to the significant association of these motifs, particularly *M26*, with DSBs in the *S. pombe* genome [18], [31], [32].

**Table 2:** Hotspot motif correlations with DSBs.

Motif^a^	number ingenome	number withinhotspots^b^	expectednumberin hotspots^c^	probability ofrandomassociation withhotspots^d^	number inintergenicDNA	expectednumber inintergenicDNA^e^	probability ofrandomassociation withintergenic DNA^d^
*M26*	26	0	2.0	0.15	10	6.2	0.084
*M26 (7 bp)*	1328	95	100	0.60	249	319	6.9×10^−6^
*CCAATCA*	1878	111	141	8.9×10^−3^	297	451	8.9×10^−17^
*Oligo-C*	35	10	2.6	2.2×10^−6^	17	8.4	6.6×10^−4^
*4095*	63	4	4.7	0.74	7	15.1	0.017
*4156*	21	7	1.6	8.2×10^−6^	12	5.0	7.8×10^−7^

a Sequence motifs are described in text. The 7 bp *M26* motif is the sequence originally identified in *S. pombe*, ATGACGT.

b hotspots as described in [5].

c based on DSBs covering 7.5% of genome [5], and an even distribution throughout.

d based on chi-squared test.

e Based on 24% of genome consisting of intergenic DNA (*Saccharomyces* Genome database. http://www.yeastgenome.org/download-data/sequence), and an even distribution throughout the genome.

Unlike the above three motifs, the *oligo-C* and *4156* motifs both showed highly significant enrichment in DSBs, indicating that these motifs may act as natural hotspots in the *S. cerevisiae* genome. Both of these motifs are enriched in intergenic DNA relative to the rest of the genome, which may be due to both containing relatively low use codons in all reading frames (data not shown). Nevertheless, this enrichment in intergenic DNA by itself is not likely to account for the significant association of these motifs with hotspots, as their association with DSBs remains significant even when considering intergenic DNA in isolation (Table 3).

**Table 3:** Significant association of *oligo-C* and *4156* motifs with DSBs in intergenic DNA.

motif	number in intergenic DNA	predicted number in DSBs^a^	observed number in DSBs	Probability^b^
*oligo-C*	17	4.9	10	6.3×10^−3^
*4156*	12	3.4	7	0.049

a Based on an intergenic DNA length of 2,852,618 bp (http://www.yeastgenome.org) and DSBs covering 28.6% of total intergenic DNA (inferred from data in Table S2 in [5]. Intergenic DNA excludes all annotated genomic features including ORF, ARS, CEN, rRNA, tRNA, snRNA, snoRNA, RNA genes, LTRs, telomeric elements, and transposons.

b Based on chi-squared test.

### Transcription Factor Dependence of Hotspots

Since there is ample precedent for hotspots requiring transcription factors [3], [7], [18], we tested whether any of the active motifs we found require specific transcription factors. Based on transcription factor binding sites reported in the literature [33], [34], we found factors that could potentially bind to three of the four active hotspots. Rds1 binds to CGGCCG, the central 6 bp of the *4156* motif. However, a knockout of *rds1* did not significantly affect recombination of that hotspot or its control (*ade2-1027* and *ade2-1034*; unpublished observation). Mig1, Mig2, and Mig3 are reported to bind CCCCGCA (seven of the eight bp *oligo-C* motif). Individual knockouts of any of these three genes had no significant effect on the hotspot or its control (*ade2-1032* and *ade2-1035*). While a triple knockout of *mig1*, *mig2*, and *mig3* reduced recombination of the *oligo-C* hotspot, it remained significantly higher than its corresponding control (unpublished observation), Thus, other factors, though not necessarily transcription factors, must be involved in making the *oligo-C* motif a hotspot.

**Figure 5:**
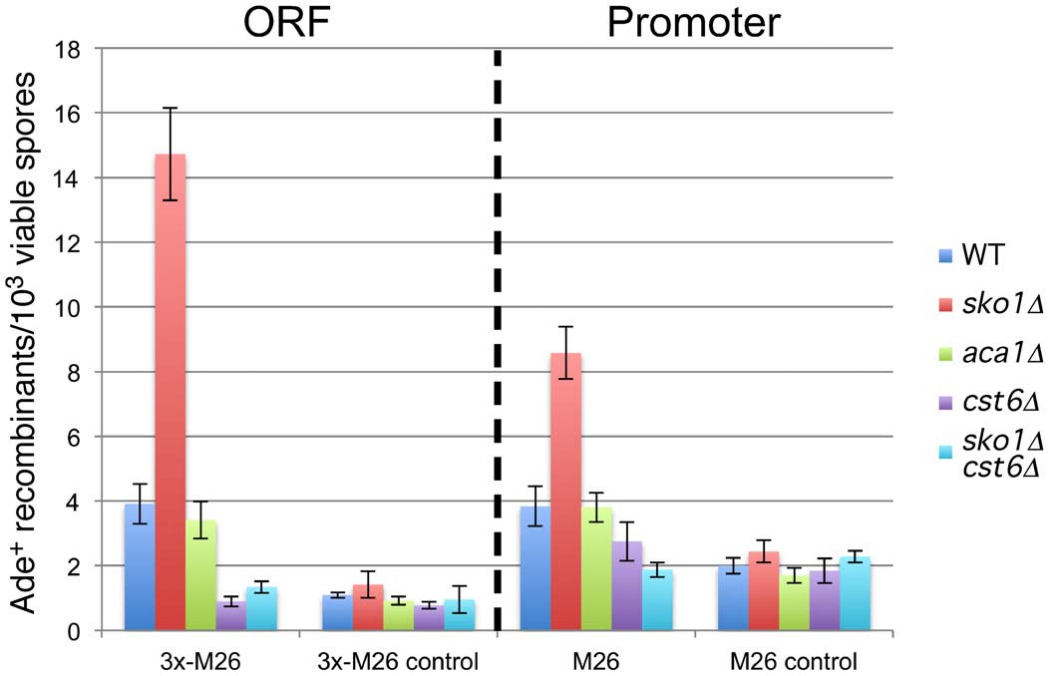
*M26* hotspot is repressed by Sko1 and activated by Cst6. Strains containing the *ade2-1003* allele and indicated transcription factor deletions were crossed to strains containing the same transcription factor deletions and the following *ade2* alleles: 3x-*M26* (*ade2-1008*; ORF), 3x-*M26* control (*ade2-1030*; ORF), *M26* (*ade2-1025*; promoter), M26 control (*ade2-1026*; promoter). Bars indicate the mean frequency of recombinants from at least three crosses. Error bars indicate one standard deviation. Transcription factor deletions are indicated by the format legend shown to the right. Crosses are from left to right: (*ade2-1008*×*ade2-1003*) Wsc70×72, Wsc126×127, Wsc184×183, Wsc157×143, Wsc179×172; (*ade2-1030*×*ade2-1003*): Wsc120×72, Wsc126×128, Wsc185×183, Wsc143×174, Wsc197×172; *(ade2-1025*×*ade2-1003*): Wsc115×72, Wsc126×129, Wsc186×183, Wsc143×144, Wsc172×173; *M26* control (*ade2-1026*×*ade2-1003*): Wsc116×72, Wsc126×130, Wsc187×183, Wsc143×145, Wsc200×172.

Given that the *M26* hotspot in *S. pombe* is known to require the heterodimeric transcription factor Atf1-Pcr1, we also tested the orthologous proteins Sko1, Cst6, and Aca1 of *S. cerevisiae*, all of which are reported to bind the *M26/CRE* motif. The strongest ortholog of both Atf1 and Pcr1 in *S. cerevisiae* is Sko1 (Acr1), a member of the ATF/CREB family of transcription factors, which binds to *CRE*-like sequences as a homodimer via a basic leucine zipper domain [35]. Sko1 is phosphorylated by the MAP kinase Hog1, which converts it from a transcriptional repressor into a transcriptional activator, and similar to Atf1, Sko1 is involved in responding to osmotic stress [36]. Surprisingly, deletion of *sko1* did not eliminate *M26* hotspot activity, but actually increased it significantly in both the promoter and the ORF (Figure 5). Consistent with it role as a transcriptional repressor, elimination of Sko1 also restored adenine prototrophy to a strain containing only the *M26* promoter substitution (*ade2-1021*; Fig. 3).

Cst6 (Aca2) and Aca1 are also bzip transcription factors with weaker homology to Atf1 and Pcr1. While loss of the Aca1 protein has no obvious phenotype, loss of Cst6 results in slow growth on glucose and poor or no growth on non-glucose carbon sources. The phenotypes associated with loss of Cst6 can be suppressed by overexpression of Aca1, suggesting functional overlap between these proteins. Both factors can bind to *M26* as either homo- or heterodimers, and both promote transcription rather than repress it [37]. Of these two genes, only deletion of *cst6* significantly reduced recombination of the *M26* hotspot in both the promoter and the ORF (Fig. 5), consistent with the observation that Aca1 may be a less active or less abundant protein than Cst6 [37]. Deletion of *cst6* was epistatic to the stimulatory effect of the *sko1* deletion (Fig. 5), which is consistent with Cst6 being essential for *M26* hotspot activity in *S. cerevisiae*. Given the reported binding specificities of Sko1 and Cst6 [37], and given that these proteins significantly affect recombination only on *ade2* alleles containing *M26*, but not sequence-matched controls (Fig. 5), it is likely that Sko1 and Cst6 affect recombination via direct binding to the *M26* motifs we created. Direct binding for Sko1 is also supported by the Sko1-dependent adenine auxotrophy that results from the presence of the *M26* motif in the promoter (Fig. 3). However, it remains formally possible that Sko1 and Cst6 affect the *M26* hotspot by a less direct mechanism.

It is interesting to note the parallel roles of Sko1 and Cst6 in repressing and activating both transcription and recombination, respectively ( [37]; Fig. 3 and Fig. 5). It is unlikely that transcription of the *ade2* gene *per se* is responsible for increasing recombination, since the *M26* motif appears to eliminate or at least greatly reduce *ade2* transcription (*ade2-1021*; Fig. 3A), even as recombination increases (Fig. 5). In addition, we noted that elimination of the native transcription factor binding sites for Bas1 and Reb1 [5], [38] also reduced or eliminated transcription of *ade2* (inferred from the adenine auxotrophy of the *ade2-1047* allele, Fig. 3B), without any significant effect on recombination (Fig. 2). This result is consistent with the previous observation that elimination of the Bas1 transcription factor also had no significant effect on the frequency of DSBs in this region [4]. Thus, we believe the most likely explanation for suppression of recombination by Sko1 is that it simply competes with Cst6 for binding of the *M26* motif, though this is speculative at this point.

### Conclusion

The results presented here are the first demonstration that sequence dependent hotspots of recombination are functional in both the fission and budding yeasts. Since these two yeasts are as evolutionarily distant from each other as either is from human beings [22], it is likely that the motifs tested here have the potential to be active in many diverse organisms with meiotic recombination hotspots. Of the four motifs with the ability to create a hotspot in *S. cerevisiae*, *M26*, *oligo-C*, *4156*, and *4095* (Fig. 2), only two actually showed a significant association with naturally-occurring hotspots in the genome (Tables 2 and 3). Thus, *S. cerevisiae*, and perhaps other organisms, have ways of preventing the activity of some sequence motifs that might otherwise act as hotspots. Although there are several factors, including local and regional chromatin structure [5], [15], that work together to determine the position of hotspots, sequence motifs are the simplest and most easily identified of these factors, and therefore may provide a useful tool for understanding and predicting the location of meiotic recombination hotpots.

## Materials and Methods

### Strain Construction

All strains used in this study are listed in Table 1 and Table S1, and are based on the A364a genetic background. Nucleotide substitutions in the *ADE2* gene were made using oligonucleotides containing the desired mutations and overlap-extension PCR as previously described [16]. Mutations were introduced into *S. cerevisiae* by transformation of Wsc55 or Wsc59 as described by Storici *et al* [39]. These strains contain a tandem insertion of the *URA3* and *kanMX4* genes, as well as a galactose-inducible I-*SceI* endonuclease and I-*Sce1* cut site. Homologous gene replacement can be stimulated by induction of a DSB and identified by the simultaneous loss of both the *URA3* and *kanMX4* markers (resistance to 5-FOA and sensitivity to G418, respectively). All transformants were verified by sequencing of the appropriate regions.

Transcription factor knockouts were obtained from EUROSCARF (http://web.uni-frankfurt.de/fb15/mikro/euroscarf/col_index.html) and were introduced into our genetic background by PCR amplification of the deleted genes and lithium acetate-mediated transformation of the appropriate strains using the protocol described in [40]. Gene knockouts were verified by PCR using a primer for *kanMX4* in combination with gene-specific primers. All primer sequences are available upon request.

### Crosses and Genetic Techniques

Crosses of haploid strains were performed on YEA-5S [25], [41] and diploids were selected on NBA (Yeast nitrogen base agar without amino acids; Difco) supplemented with 10 µg/ml adenine, 50 µg/ml uracil, and 50 µg/ml histidine. Individual colonies of diploid strains were grown overnight in 5 ml YPD medium [42] supplemented with 100 µg/ml adenine. Saturated cultures were washed once in 10 ml H_2_O, then resuspended in 10 ml sporulation medium (2% potassium acetate, 0.1% yeast extract, 0.05% glucose, 100 µg/ml adenine, 50 µg/ml uracil, 50 µg/ml histidine). Sporulation was performed at 30°C with aeration for 3–5 days. In order to minimize potential differences in recombination frequencies due to factors other than nucleotide sequence, i.e. day-to-day variation, motifs and their respective controls were always tested side-by-side in sets of three to five cultures each.

Sporulated cultures were washed once in 10 ml H_2_O, then resuspended in one ml H_2_O. Intact asci were plated to NBA dropout medium [42] lacking arginine and containing 10 µg/ml cycloheximide and 60 µg/ml canavanine (to titer viable asci) or lacking arginine and adenine (to titer Ade^+^ recombinants). Since the *can1* and *cyh2* mutations carried in one parent of each cross are recessive alleles conferring resistance to canavanine and cycloheximide, respectively, unsporulated diploid cells are unable to grow on this medium ( [43] and our observation). However, one-fourth of spores (two spores in one-half of asci) become resistant to both drugs following meiosis. The use of intact asci in these experiments is likely to overestimate the actual frequency of recombinants about two-fold compared to disrupted asci, but would not significantly change the relative differences between strains.

### Correlation of DSBs and Motifs

The location of particular motifs in the *S. cerevisiae* genome was determined using the pattern matching function available through the *Saccharomyces* genome database (http://www.yeastgenome.org/). The location of motifs located exclusively in intergenic regions was determined by using the pattern matching function on the "NotFeature" portion of the genome, which excludes all protein-coding and RNA genes, and several other annotated features. These intergenic sequences comprise approximately 24% of the sequenced genome.

## Supporting Information

Table S1 **Strains and** ***ADE2*** **allele descriptions.**
(DOCX)
