# Supplementary material for: Fission Yeast Hotspot Sequence Motifs Are Also Active in Budding Yeast
Source: PLoS One. 2012 Dec 31;7(12):e53090. doi: 10.1371/journal.pone.0053090 (PMC3534124; doi:10.1371/journal.pone.0053090)
Supplement: Table S1 — Strains and ADE2 allele descriptions. (DOCX) [file pone.0053090.s001.docx]

Table S1. Strains and *ADE2* allele descriptions.

| **Strain number** | ***ADE2* allele** | **Mating type** | **description^a^** | **Nucleotide sequence^b^** | **position^c^** |
| --- | --- | --- | --- | --- | --- |
| Wsc24 | *+* | **a** | wild-type | N.A. | N.A. |
| Wsc55 | 1004 | **a** | *ura3Kl-kanMX4* | N.A. | 95^d^ |
| Wsc59 | 1005 | α | *ura3Kl-kanMX4* | N.A. | Δ96-1120^e^ |
| Wsc64 | 1006 | **a** | *M26* | A**T**GACG**TC**A**T** | 82-91 |
| Wsc66 | 1007 | **a** | 5'-stop | **T**AA | 103-105 |
| Wsc70 | 1008 | **a** | 3x-*M26* | (ATGACGTCAT)_3_ | 82-111 |
| Wsc72 | 1003 | α | 3'-stop | **T**AA | 1111-1113 |
| Wsc92 | 1014 | **a** | *CCAAT* | **CC**GACC**AA**T**C**ATA**T** | 82-94 |
| Wsc94 | 1015 | **a** | *Oligo-C* | AGA**AC**C**CCGC**A | 82-91 |
| Wsc97 | 1016 | **a** | *4095* | **GGTCTG**G**AC**C | 81-89 |
| Wsc103 | 1017 | **a** | *4156* | T**C**G**G**C**C**G**A** | 83-89 |
| Wsc110 | 1021 | **a** | *M26* (no 5'-stop) | ATG**ACGTC**AT | -(98-89) |
| Wsc115 | 1025 | **a** | *M26* | ATG**ACGTC**AT | -(98-89) |
| Wsc116 | 1026 | **a** | *M26* control | ATG**GTACC**AT | -(98-89) |
| Wsc117 | 1027 | **a** | *4156* | **AA**T**TCG**G**CCG**A**G**A**C** | -(102-89) |
| Wsc118 | 1028 | **a** | *CCAAT* | **G**T**CCTCCAATC**AA**T**T | -(103-89) |
| Wsc120 | 1030 | **a** | 3x-*M26* control | (ACTAGCTAGT)_3_ | 82-111 |
| Wsc121 | 1031 | **a** | *4095* | **GGTCTGG**A**CC** | -(98-89) |
| Wsc125 | 1032 | **a** | *oligo-C* | **G**A**ACCCCGC**A**C** | -(99-89) |
| Wsc126 | 1003 | α | 3'-stop *sko1Δ* | **T**AA | 1111-1113 |
| Wsc127 | 1008 | **a** | 3x-*M26* *sko1Δ* | (ATGACGTCAT)_3_ | 82-111 |
| Wsc128 | 1030 | **a** | 3x-*M26* control *sko1Δ* | (ACTAGCTAGT)_3_ | 82-111 |
| Wsc129 | 1025 | **a** | *M26 sko1Δ* | ATG**ACGTC**AT | -(98-89) |
| Wsc130 | 1026 | **a** | *M26* control *sko1Δ* | ATG**GTACC**AT | -(98-89) |
| Wsc134 | 1034 | **a** | *4156* control | **AAGT**A**G**G**CG**T**C**A**CC** | -(102-89) |
| Wsc135 | 1035 | **a** | *oligo-C* control | **G**A**CCACC**A**GCC** | -(99-89) |
| Wsc136 | 1036 | **a** | *oligo-C* control | G**CC**AC**CAGC** | 83-90 |
| Wsc143 | 1003 | α | 3'-stop *cst6Δ* | **T**AA | 1111-1113 |
| Wsc144 | 1025 | **a** | *M26 cst6Δ* | ATG**ACGTC**AT | -(98-89) |
| Wsc145 | 1026 | **a** | *M26* control *cst6Δ* | ATG**GTACC**AT | -(98-89) |
| Wsc156 | 1038 | **a** | *4095* control | **GTCGGTCGC** | -(97-89) |
| Wsc157 | 1008 | **a** | 3x-*M26* *cst6Δ* | (ATGACGTCAT)_3_ | 82-111 |
| Wsc166 | 1047 | **a** | Bas1/Reb1 BSKO^f^  (no stop) | **AGT**…**AAC**…**AGT** | -(195-193…169-167…152-150) |
| Wsc167 | 1048 | **a** | *CCAAT* control | **TTAC**TC**TCA**T**GC**A**C**A**C** | -(104-89) |
| Wsc168 | 1049 | **a** | Bas1/Reb1 BSKO^f^ | **AGT**…**AAC**…**AGT** | -(195-193…169-167…152-150) |
| Wsc169 | 1050 | **a** | *M26* control | **T**C**T**G**CT** | 85-90 |
| Wsc172 | 1003 | α | 3'-stop *cst6Δ* *sko1Δ* | **T**AA | 1111-1113 |
| Wsc173 | 1025 | **a** | *M26 cst6Δ sko1Δ* | ATG**ACGTC**AT | -(98-89) |
| Wsc174 | 1030 | **a** | 3x-*M26* control *cst6Δ* | (ACTAGCTAGT)_3_ | 82-111 |
| Wsc179 | 1008 | **a** | 3x-*M26* *cst6Δ* *sko1Δ* | (ATGACGTCAT)_3_ | 82-111 |
| Wsc183 | 1003 | α | 3'-stop *aca1Δ* | **T**AA | 1111-1113 |
| Wsc184 | 1008 | **a** | 3x-*M26* *aca1Δ* | (ATGACGTCAT)_3_ | 82-111 |
| Wsc185 | 1030 | **a** | 3x-*M26* control *aca1Δ* | (ACTAGCTAGT)_3_ | 82-111 |
| Wsc186 | 1025 | **a** | *M26 aca1Δ* | ATG**ACGTC**AT | -(98-89) |
| Wsc187 | 1026 | **a** | *M26* control *aca1Δ* | ATG**GTACC**AT | -(98-89) |
| Wsc197 | 1030 | **a** | 3x-*M26* control *sko1Δ cst6Δ* | (ACTAGCTAGT)_3_ | 82-111 |
| Wsc200 | 1026 | **a** | *M26* control *sko1Δ cst6Δ* | ATG**GTACC**AT | -(98-89) |
| Wsc202 | 1021 | **a** | *M26* (no 5'-stop) *sko1Δ* | ATG**ACGTC**AT | -(98-89) |

^a^Name of hotspot motifs and control alleles in *ADE2* gene and transcription factor (TF) gene knockouts, if present. TF gene knockouts are insertion-deletions that substitute *kanMX4* for the coding sequence. In addition to the listed mutations, all Mat **a** strains are also *trp1-289 ura3-52 his7* and all Mat α strains are also *leu2-3,112 ura3-52 his7 can1 cyh2*. In addition, all *ade2* alleles except *ade2-1003, -1004, -1005, -1008, and -1030* contain the same stop mutation as the *ade2-1007* allele unless otherwise indicated (no stop). *ade2-1008* and *-1030* contain different stop mutations incorporated in their sequence substitutions.

^b^The relevant sequence of *ADE2* is shown. Nucleotide substitutions are shown in boldface type. Nucleotide insertions are underlined. The *ade2-1008* and *ade2-1030* alleles contain multiple substitutions, which are not indicated, and no insertions. N.A. Not applicable.

^c^The position of the sequence shown in the proceeding column, with the first nucleotide of the coding sequence considered as +1 and the preceding nucleotide as -1. Insertions are not counted.

^d^An insertion of the *Gal1-I-SceI kanMX4 KlURA3* cassette after nucleotide 95 of *ADE2*. This construct allows for a galactose-inducible double-strand break within the cassette to facilitate the introduction of *ADE2* sequence substitutions by homologous recombination ([32](#_ENREF_32)).

^e^*ADE2 Δ*96-1120 with insertion of the *Gal1-I-SceI kanMX4 KlURA3* cassette.

^f^Binding Site KnockOuts.
